# Supplementary material for: Characterization of the external exposome and its contribution to the clinical respiratory and early biological effects in children: The PROMESA cohort study protocol
Source: PLoS One. 2023 Jan 20;18(1):e0278836. doi: 10.1371/journal.pone.0278836 (PMC9858469; doi:10.1371/journal.pone.0278836)
Supplement: Attachment — Submitted filename: The PROMESA Cohort Study Protocol Review.docx [file pone.0278836.s002.docx]

**Re Characterization of the External Exposome and its Contribution to the Clinical Respiratory and Early Biological Effects in Children: The PROMESA Cohort Study Protocol**

Comments for the authors

ABSTRACT

- Specify if this work is focused on ambient (outdoor) vs. indoor exposures.
- Specify if asthma diagnosis is physician-based and if standardized protocol is used

INTRODUCTION

- Generally, the references used are not focused on the population of interest. For example, it would be a better fit to provide the global burden of disease among pediatric population.
- Health effects references line 89 are mostly for the adult population, suggest revising the used references
- I would referring to work on genetic clock, telomere lengths and air pollution in early life. For example
  - Isaevska E, Moccia C, Asta F, Cibella F, Gagliardi L, Ronfani L, Rusconi F, Stazi MA, Richiardi L. Exposure to ambient air pollution in the first 1000 days of life and alterations in the DNA methylome and telomere length in children: A systematic review. Environmental research. 2020 Nov 20:110504.
  - de Prado-Bert P, Ruiz-Arenas C, Vives-Usano M, Andrusaityte S, Cadiou S, Carracedo Á, Casas M, Chatzi L, Dadvand P, González JR, Grazuleviciene R. The early-life exposome and epigenetic age acceleration in children. Environment International. 2021 Oct 1;155:106683.

MATERIAL AND METHODS

- It is debatable whether this is a prospective cohort or a retrospective birth cohort of the first 5 years of life. I would argue that it is the latter given the assessment of exposure prenatally and that the prospective work is only for 1 year.
- More details are needed re: Health Insurance Company. Is this a national health coverage system? If not, would this requirement for the participants selection lead to biases? Is Colombia’s health care system a two tier system? More generally what does this criterion imply?
- Line 196: what is the definition of prematurity? How is gestational age assessed?
- I would expect that only singleton births would be included – Why is this not the case here?

OBJECTIVES AND OUTCOMES MEASUREMENTS

- A major limitation is the fact that asthma diagnosis at age 5 is usually complex. The authors have provided a national guideline document, but (1) it is in Spanish; (2) it is unclear if this is valid for age 5 years,
- Line 225: given the evolution of the pandemic, do authors truly believe that the timeline for recruitment is feasible?

LABORATORY EVALUATIONS, ASSAYS, and HANDLING

- What type of selection bias would the authors expect from the selection of these list of genes?
- Lines 268-271: how will the sampling sites be chosen? What risks associated with high volume samples over 2 weeks in heavily polluted areas and conversely with low volume samplers in "cleaner" areas?

SAMPLE SIZE SELECTION and STATISTICAL ANALYSES

- RR chosen by the authors is based on the ESCAPE project publications, this is for upper ARI based and for likely lower exposure levels that what would be expected in the study sites of the proposed research protocol. Therefore, I would expect a much lower effect estimate of lower ARI and asthma. Various simulations (high, medium, and low exposure levels along with high and low RR) would allow a range of sample size to consider.
- Line 332: how are the authors planning to measure surrounding natural spaces?
- Lines 341 to 343: this is quite vague and presents different approaches with little information on how the modeling will be conducted.

DISCUSSION

- One of the major limitations of this work is the contribution of the indoor exposures and the fact that all exposures are based on residential home address. This should be addressed/commented on in the discussion
